# Robust Colloidal Synthesis of Palladium–Gold Alloy Nanoparticles for Hydrogen Sensing

**DOI:** 10.1021/acsami.1c15315

**Published:** 2021-09-20

**Authors:** Sarah Lerch, Alicja Stolaś, Iwan Darmadi, Xin Wen, Michał Strach, Christoph Langhammer, Kasper Moth-Poulsen

**Affiliations:** † Department of Chemistry and Chemical Engineering, Chalmers University of Technology, SE-412 96 Gothenburg, Sweden; ‡ Department of Physics, Chalmers University of Technology, SE-412 96 Gothenburg, Sweden; § Chalmers Materials Analysis Laboratory, Chalmers University of Technology, SE-412 96 Gothenburg, Sweden

**Keywords:** metal nanoparticles, nanoparticle synthesis, colloidal synthesis, hydrogen, palladium−gold alloys, sensors

## Abstract

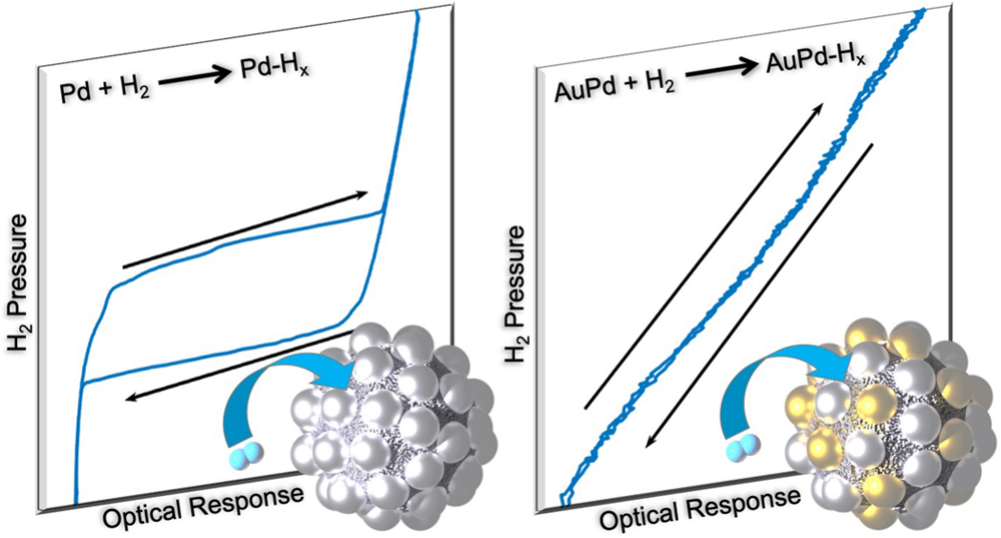

Metal nanoparticles are currently used in a variety of applications, ranging from life sciences to nanoelectronic devices to gas sensors. In particular, the use of palladium nanoparticles is gaining increasing attention due to their ability to catalyze the rapid dissociation of hydrogen, which leads to an excellent response in hydrogen-sensing applications. However, current palladium-nanoparticle-based sensors are hindered by the presence of hysteresis upon hydride formation and decomposition, as this hysteresis limits sensor accuracy. Here, we present a robust colloidal synthesis for palladium–gold alloy nanoparticles and demonstrate their hysteresis-free response when used for hydrogen detection. The obtained colloidal particles, synthesized in an aqueous, room-temperature environment, can be tailored to a variety of applications through changing the size, ratio of metals, and surface stabilization. In particular, the variation of the viscosity of the mixture during synthesis resulted in a highly tunable size distribution and contributed to a significant improvement in size dispersity compared to the state-of-the-art methods.

## Introduction

Since the identification of the unique optical properties of metallic nanoparticles (NPs) by Michael Faraday,^1,2^ there have been numerous studies of the physics behind this distinct optical response. These optical properties are dominated by the localized surface plasmon resonance (LSPR),^3−5^ which is highly sensitive to the size, shape, and composition of the NPs, as well as the environment surrounding them.^6−9^ This exceptional tunability of the optical response has led to the attempted use of plasmonic NPs in a variety of applications. Successful examples range from life sciences to catalysis and bio/gas sensing and involve a large variety of metal or semiconducting NPs with different shapes, sizes, and compositions, obtained with different synthesis techniques.^10−13^ In the previous decade, there has also been increasing interest in improving the ability to customize the properties of NPs by combining multiple metals and oxides, either by linking together multiple single-metal NPs or by alloying the metals at the atomic level to form alloyed NPs.^14−18^ To this end, the combination of noble metals with other elements can create increased reactivity for catalysis and introduce significant lattice strain on the atomic scale, which has positive implications for both catalysis and sensing.^19−22^ Accordingly, there are numerous successful applications of alloyed NPs, including the use of gold–silver (AuAg) alloyed NPs for the sensing of various organic and biological compounds and the use of palladium–gold (PdAu),^22−24^ palladium–silver (PdAg),^25^ and even palladium–gold–copper (PdAuCu) NPs^16,70^ for hydrogen (H_2_) sensing, storage, and catalysis.

The use of Pd-based alloyed NPs for H_2_ sensing is an area of current interest due to the importance of renewable and safe energy technologies to combat climate change. Hydrogen-based energy systems have the potential to be incorporated as a crucial part of the solution if certain concerns can be addressed, one of which being the necessity of fast, robust, and cost-efficient safety sensors for the detection of leaks or sudden pressure changes. In this context, Pd is very attractive due to the barrierless dissociation of H_2_ molecules on the surface of Pd metal, provided that the surface is free from contamination.^26^ Furthermore, once dissociated, hydrogen atoms (H) can occupy interstitial lattice sites in the Pd host to form a solid solution at low hydrogen concentration and a hydride above a critical concentration.^27^ Both solid solution and hydride formation lead to the change of the Pd electronic structure and volume and, thus, a change in both its electronic and optical responses.^24,28^ Focusing on optical properties, in Pd NPs, this results in a distinct shift of the LSPR, allowing for sensitive optical detection of the presence of hydrogen in the environment.^29−31^ However, one of the main limitations of hydrogen sensors using pure Pd, whether bulk, thin films, or NPs, is the inherent hysteresis between hydride formation and decomposition, which dramatically reduces sensor accuracy in the pressure range where hysteresis occurs.^32−34^ The aforementioned alloyed NPs, specifically those of PdAg, PdAu, and PdAuCu, eliminate this hysteresis while retaining the key traits of Pd with respect to hydrogen sensing in general, and for plasmonic hydrogen sensing in particular. Notably, however, the development of hysteresis-free plasmonic hydrogen sensors based on Pd–noble metal alloy nanoparticles has taken place almost exclusively with structures fabricated using nanolithography and thin film deposition techniques, which are somewhat limited in scalability. Therefore, we predict an increasing interest in solutions where such alloy NP systems can, instead, be produced by cost-effective and scalable colloidal synthesis. Such an approach will, in addition, enable the production and use of smaller nanostructures, which is beneficial from a sensor response time perspective due to reduced H diffusion paths lengths,^24,35^ and enables tapping into surface/volume ratio-related effects.^9,12,36^ To this end, while there are several published methods for the synthesis of PdAu or PdAg alloy colloidal NPs, the synthesis of bimetallic alloys remains challenging since commonly used techniques, such as electrolysis, demand high precision in, for example, chemical reagents, resulting in significant experiment-to-experiment variation.^14,37−40^ Additionally, there are multiple potential issues that need accurate control, such as (1) different reduction rates of metals, resulting in core–shell structures, (2) selection of suitable stabilizing agents, allowing for the formation of aggregate-free nanostructures, and (3) selection of a proper reducing agent to allow an appropriate time for alloy growth.

In response, here, we present a reproducible synthesis for colloidal PdAu alloy NPs that covers nearly the entire spectrum of metal ratios, from 90% Pd to 90% Au. This allows us to tailor this colloidal system toward a variety of possible sensing or catalytic applications. Further, we have thoroughly characterized these NPs using grazing incidence X-ray powder diffraction (GIXRD), high-resolution transmission electron microscopy (HRTEM), selected-area electron diffraction (SAED), and energy-dispersive X-ray spectroscopy (EDX), all together confirming the formation of fully and homogeneously alloyed NPs. Finally, we have tested several of the synthesized NP systems in proof-of-principle H_2_-sensing experiments to determine the optimal Pd:Au ratio for H_2_ detection and verify the anticipated suppression of hysteresis between hydride formation and decomposition.

## Experimental Section

### Chemicals

Gold(III) chloride trihydrate (HAuCl_4_·3 H_2_O, ≥99.9%, Aldrich), sodium tetrachloropalladate (Na_2_PdCl_4_, 98%, Sigma-Aldrich), l-ascorbic acid (≥99%, Sigma-Aldrich), polyvinylpyrrolidione (PVP, average Mw ∼ 55 000, Aldrich), hexadecyltrimethylammonium bromide (CTAB, ≥99%, Sigma-Aldrich), polyethylenimine (PEI, branched, average Mw ∼ 25 000, Sigma-Aldrich) sodium bromide (NaBr, ≥99%, Sigma-Aldrich), potassium chloride (KCl, ≥99%, Sigma-Aldrich), and ethylene glycol (AnalaR NORMAPUR, VWR) were used without further purification. Ultrapure water (Milli-Q Advantage A10 water purification, Merck) was used for all nanoparticle synthesis and washing.

### Colloidal Synthesis of PdAu Alloy Nanoparticles

The synthesis of PdAu alloy nanoparticles, adapted from the work of Yuan et al.,^38^ begins with dissolving a mixture of HAuCl_4_ and Na_2_PdCl_4_ salts in Milli-Q water, according to Table S1 (Supporting Information, SI), such that the total concentration of metals was 20 mM. Separately, 47 mL of Milli-Q water was added to a 100 mL round-bottom flask suspended in air above a stir plate. A medium stir bar was added to the flask and the contents of the flask were stirred at 500 rpm, or other speeds as indicated. One milliliter of the HAuCl_4_/Na_2_PdCl_4_ solution was added to the flask and stirred until the color was light yellow or brown, depending on the Pd:Au ratio, and evenly distributed. One milliliter of l-ascorbic acid (100 mM) was added to the stirred solution, resulting in an immediate color change to dark brown or black. After approximately 10 s, 1 mL of PVP solution (5 mg/mL) was added to the solution with stirring. The solution was stirred for 30 min to facilitate growth of the NPs. The NP colloidal solution was then centrifuged for 5 min at 10 000 rpm and further washed two times with Milli-Q water to remove excess PVP. The solution was then redispersed in 3 mL of Milli-Q water.

### X-ray Powder Diffraction in Grazing Incidence (GIXRD)

GIXRD patterns were acquired using a SAXSLAB Mat:Nordic instrument equipped with a microfocus Cu X-ray source and Dectris Pilatus 300 K R (low angle 2θ ≤ 30°) and 100 K R (27° ≤ 2θ ≤ 85°) detectors. The entire beam path was evacuated to 0.15 mbar before each measurement to minimize air scattering at low angles. The position and tilt of the sample were calibrated before each measurement. Patterns were acquired at 1° incidence angle to maximize the signal from the deposited material. The peak positions and instrument broadening were assessed using a NIST-certified corundum powder measured in the same configuration as the sample. Rietveld refinement^41^ was performed using Bruker TOPAS V6 software, taking a pure Au structure as a starting point (cubic *Fm*3̅*m* PDF 00-004-0884). The model included several corrections: displacement correction, emission profile, polynomial background, spherical harmonics, instrumental peak shape (Simple_axial_model and Pearson 7), and size broadening. Average crystallite radius was determined from the volume-weighted mean column height calculated by the macro LVol in TOPAS.

Samples were prepared on 1 × 1 cm single-crystal silicon wafers that were pretreated with an oxygen plasma (30 s). First, a solution of 0.01% poly-l-lysine was drop-cast on the sample surface for 10 min. This solution introduced a thin polymer layer that allows for the NPs to efficiently stick to the Si surface.^42^ After washing away the poly-l-lysine solution with water and blow-drying with N_2_ gas, the NP solution was then drop-cast on the treated sample surface and left in a saturated environment overnight. The excess NP solution was washed away, and the sample was blow-dried with N_2_ gas (Figure S6, SI).

### Scanning Electron Microscopy (SEM)

SEM images were obtained on a Leo Ultra 55 with a field emission gun and CCD. Samples were prepared following the same method as the samples for GIXRD analysis.

### Transmission Electron Microscopy (TEM)

Lower magnification (<300 k×) TEM imaging was performed on a FEI Tecnai T20 with a LaB6 filament and Orius CCD, operated at 200 kV. Samples were prepared on copper TEM grids with carbon films (Ted Pella) by drop-casting a 2–5 μL drop of NP solution and drying in air for at least 30 min.

### Scanning Transmission Electron Microscopy (STEM)

STEM imaging, EDX measurements, and higher magnification (>300 k×) TEM imaging were performed on a FEI Titan with a field emission gun, monochromator, US1000CCD, and Oxford X-sight EDS, operated at 300 kV. Samples were prepared following the method for TEM samples.

### Hydrogen Pressure–Optical Response Isotherms

Pd NPs were synthesized as a control for hydrogen pressure–optical response isotherms. Adapted from a standard PVP-stabilized Pd nanocube synthesis,^43^ PVP (105 mg), l-ascorbic acid (60 mg), and Na_2_PdCl_4_ (57 mg) were dissolved in 11 mL of Milli-Q water in a 50 mL round-bottom flask. The solution was then heated in a 90 °C oil bath with magnetic stirring (700 rpm) for 3 h and cooled to room temperature. The resulting Pd NPs were centrifuged at 10 000 rpm for 30 min and washed two times to remove excess PVP (Figure S13, SI). The Au nanoplasmonic sensor was nanofabricated using the hole-mask colloidal lithography method published elsewhere.^44^ The sensor consists of a quasi-random array of Au nanodisks of 20 nm height and 100 nm average diameter on a 1 × 1 cm fused-silica support. The Au nanodisk array is coated with a 10 nm SiN*_x_* layer deposited by means of PECVD (plasma-enhanced chemical vapor deposition).^45^ As with the GIXRD and SEM samples, a solution of 0.01% poly-l-lysine was drop-cast on the sensor surface for 10 min. After washing away the poly-l-lysine solution with water and blow-drying with N_2_ gas, the NP solution was then drop-cast on the treated sensor surface and left in a saturated environment overnight. The excess NP solution was washed away, and the sample was blow-dried with N_2_ gas.

The hydrogen pressure–optical response isotherm measurement was performed in a homemade vacuum chamber equipped with a custom temperature controller and two capacitance manometers (MKS Barathron 626C) of different pressure ranges (the setup schematic is available elsewhere^46^). The isotherm was measured at 30 °C. The hydrogen pressure in the chamber is controlled by leak valves. The chamber has two vacuum-grade fused-silica viewports placed on opposite sides that enable transmission-mode optical measurements. One viewport is connected to a light source (Avantes AvaLight-Hal), and the other one to a fixed-grating spectrophotometer (Avantes SensLine AvaSpec-2048XL). Prior to each optical isotherm measurement, the sample is exposed to 10 cycles of 500 mbar of hydrogen at 30 °C to activate the sample and stabilize its response. Stable response is usually achieved by the fifth cycle. The full width of half-maximum (fwhm) of the LSPr peak used as the readout parameter was obtained by fitting a Lorentzian to the peak.

## Results and Discussion

**Figure 1:**
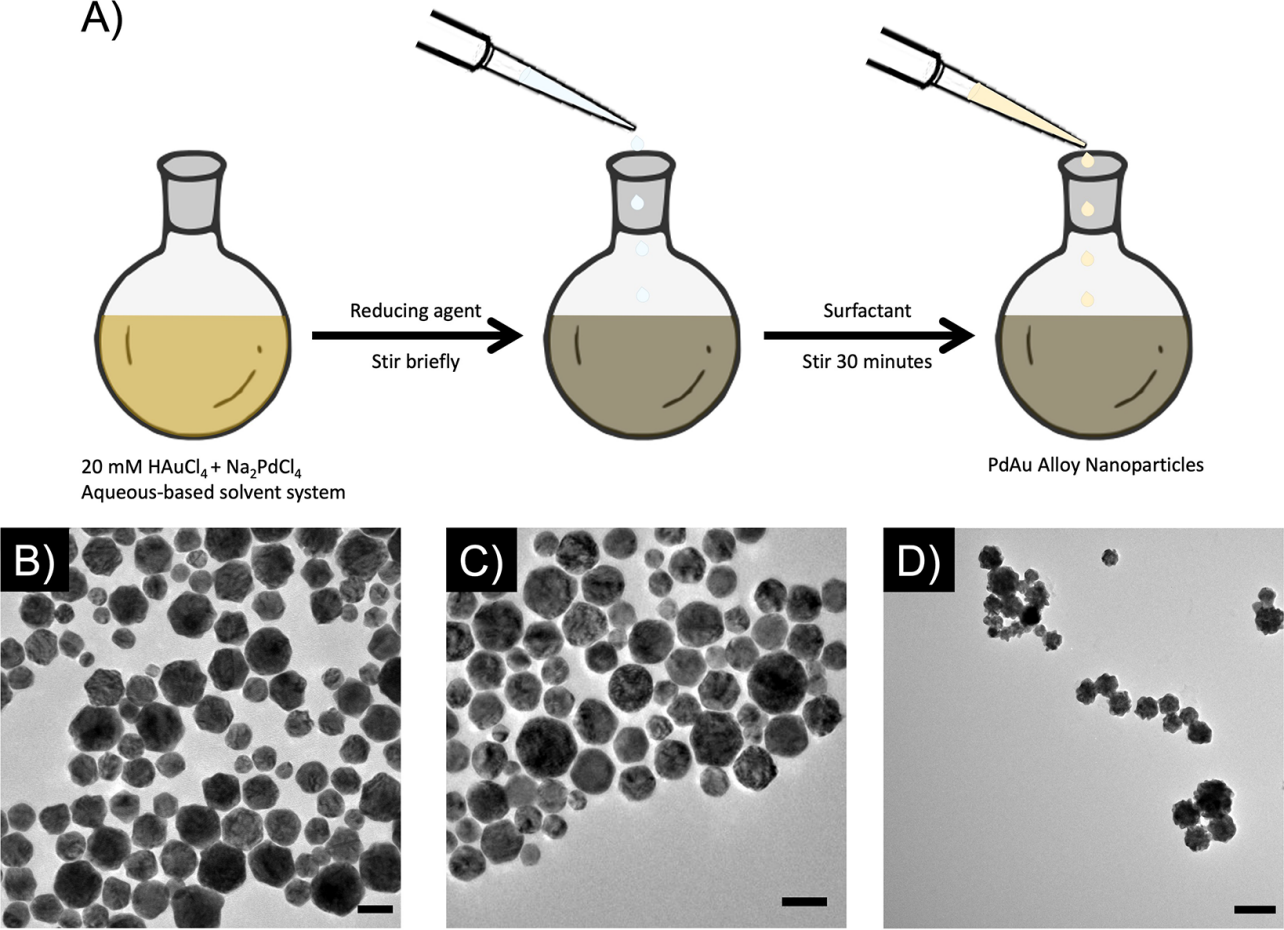
Synthesis of palladium–gold (PdAu) alloy nanoparticles (NPs). (A) Schematic showing the simple, room-temperature synthesis of 50 nm-Pd_*x*_Au_*y*_ alloy nanoparticles, where *x* and *y* are determined by the ratio of HAuCl_4_ and Na_2_PdCl_4_ in the initial mixture. Images of (B) 50 nm-Pd_7_Au_3_ nanoparticles stabilized with polyvinylpyrrolidone (PVP), (C) 50 nm-Pd_7_Au_3_ nanoparticles stabilized with hexadecyltrimethylammonium chloride (CTAB), and (D) 50 nm-Pd_7_Au_3_ nanoparticles stabilized with polyethylenimine (PEI).

The colloidal synthesis of Pd–Au alloy NPs occurs as a one-step reduction and growth, as shown in Figure 1A. This one-step method maintains the ratio of Pd:Au throughout the colloidal suspension, as well as in individual particles, leading to a robust, reproducible response based on the initial Pd:Au ratio. Furthermore, contrary to traditional NP synthesis, the metallic salts must be reduced prior to the addition of stabilizing surfactants or polymers, as these stabilizers can isolate the metals from each other (Figure S1, SI). The color of the final solution is dependent on the ratio of metals in the alloy NPs and results in a colloid with high polycrystallinity and polydispersity with an average size of 47 ± 14 nm [Figures 1B–D and S2 (SI)]. This method is applicable for Pd:Au ratios ranging from 1:9 to 9:1, and the final metal composition within the NPs is comparable to the ratio introduced during the synthesis (Figure S2, SI). We observed that the actual amount of Au is slightly higher compared to the expected nominal composition. This occurs because Au is a more noble metal when compared to Pd, so the Au^3+^ ions are reduced more rapidly than the Pd, and the Au content is therefore slightly increased in the NPs. We will refer to the alloy NPs by their diameter (in nm) and synthesis ratios (i.e., Pd_7_Au_3_ or 50 nm-Pd_7_Au_3_ for NPs with an approximate diameter of 50 nm) in this work.

We observed reproducible results with both positively charged (hexadecyltrimethylammonium bromide/chloride, CTAB/C; polyethylenimine, PEI) and negatively charged (polyvinylpyrrolidone, PVP) stabilizers reduced by ascorbic acid, as shown in Figure 1B (PVP) and Figure 1C,D (CTAB, PEI) for Pd_7_Au_3_ NPs. The addition of excess halide ions in the initial metallic salt solution (of the same ratio) did not result in significant shape transformation due to the high level of polycrystallinity present in the colloids (Figure S3, SI). Changes in temperature and stirring speed also resulted in NPs with diameters of approximately 50 nm, with similar polydispersity and polycrystallinity as in the above cases (Figure S4, SI). From those observations and the variety of alterations we made without significantly changing the resulting particles, we conclude that this method is highly reproducible.

**Figure 2:**
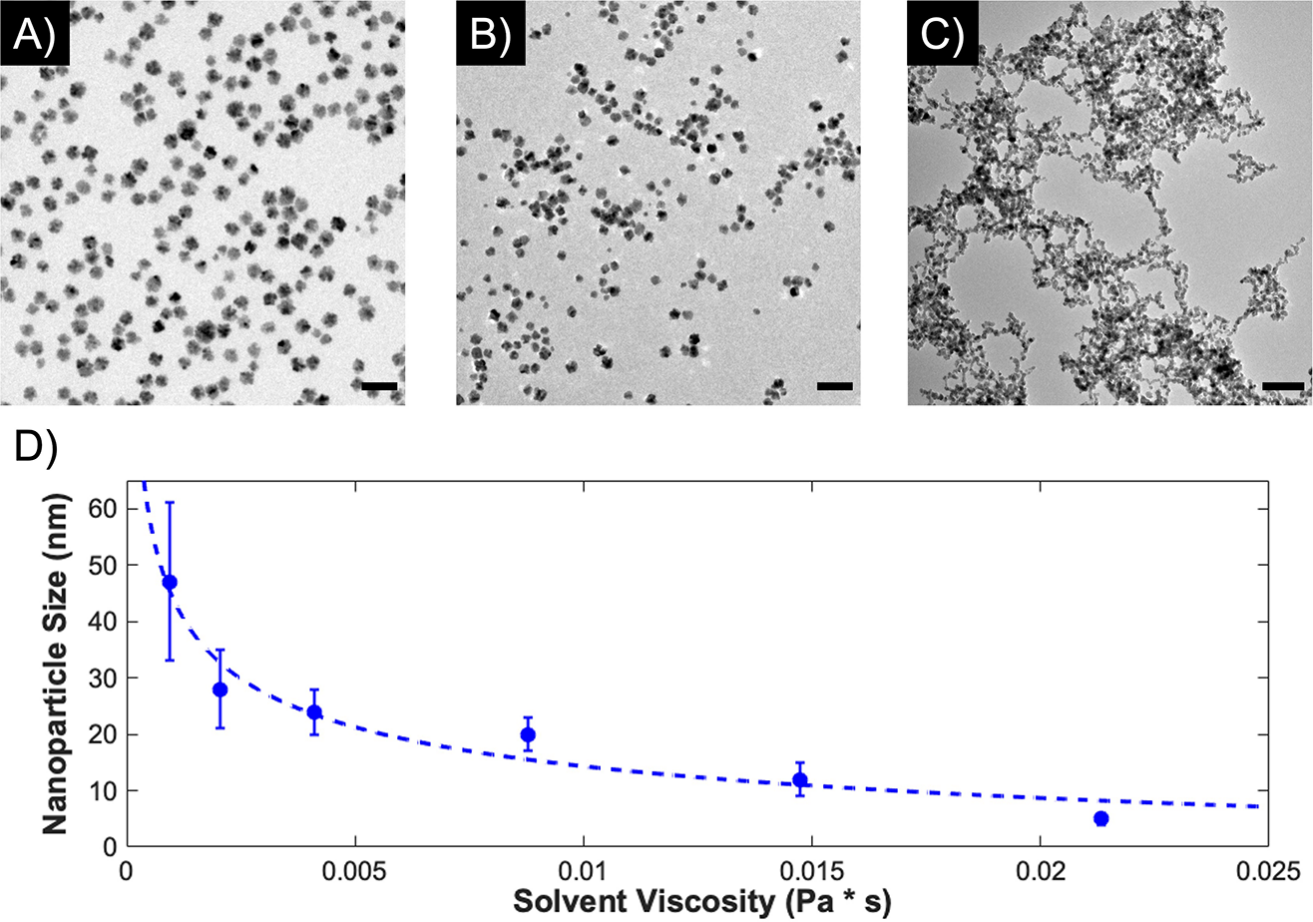
Effect of increasing solvent viscosity on particle size and distribution through addition of ethylene glycol to the solvent mixture. (A) TEM image of Pd_7_Au_3_ particles, synthesized in 75% ethylene glycol, 25% water. (B) TEM image of Pd_7_Au_3_ particles, synthesized in 90% ethylene glycol, 10% water. (C) TEM image of Pd_7_Au_3_ particles synthesized in 100% ethylene glycol, heated to 100°C. Scale bars for TEM images in panels A–C are 50 nm. (D) Relationship of NP size with the increasing viscosity provided by the ethylene glycol. Synthesis in pure water, shown in Figure 1B–D, results in a viscosity of 0.00096 Pa*s. Additions of ethylene glycol included 25% (Figure S5A, 0.0021 Pa*s), 50% ethylene glycol (Figure S5B, 0.0041 Pa s), 75% (Figure 2A, 0.0088 Pa s), and 90% (Figure 2B, 0.015 Pa*s). Synthesis in pure ethylene glycol (Figure 2C, 0.021 Pa*s) resulted in aggregated networks of small particles.

While these adjustments demonstrated the high level of reproducibility associated with our modified synthesis method, there is also significant interest in both reducing polydispersity and changing the size of these NPs. Therefore, we focused on PdAu NPs stabilized by PVP for further testing and characterization. The use of PVP, in particular, is important for application purposes in the hydrogen sensor context, since it has been demonstrated that PVP does not impede the movement of H_2_ from the environment to the surface of the Pd, unlike the other common NP surfactants that we tested.^47^ One potential method for adjusting the size of the colloidal NPs is changing the viscosity of the solution, which can change the interactions between the seed particles that are formed when the reducing agent is introduced.^48−51^ Therefore, we attempted to reproduce the synthesis of Pd_7_Au_3_ NPs in mixtures of ethylene glycol and water. When the ratio of ethylene glycol to water is increased, the obtained alloy NPs exhibit increasingly higher monodispersity and smaller size, while keeping a similar polycrystalline morphology [Figures 2A,B and S5 (SI)]. As the percentage of ethylene glycol is increased to 75%, the size of the NPs decreased to 20 ± 3 nm (Figure 2A). When the percentage of ethylene glycol is increased further to 90%, the size continues to decrease to 12 ± 3 nm (Figure 2B), but the significantly increased viscosity and small size make the recovery of the NPs difficult, whether through centrifugation or flocculation. The measured viscosity due to the increase in ethylene glycol content during the synthesis and the NP size and distributions are reported in Figure 2D, showing an excellent agreement between the decreased size and the increasing viscosity. Additionally, we observed the same trend for the composition of these alloys (Figure S5, SI) as previously noted, namely, that there is a slight increase in the Au content from the expected theoretical value but that, overall, the increasing ratio of Pd:Au matches with the molar ratio of the metallic salts added during the synthesis.

Since ethylene glycol is also a known reducing agent,^52^ we investigated the effect of the ethylene glycol beyond the increased viscosity by using ethylene glycol as the reductant and solvent at the same time.^51,53−55^ In order to increase the effectivity of the reduction via ethylene glycol, the solution was heated to 100 °C prior to the addition of the metal salts. At this temperature, the metallic ions, added in a solution of ethylene glycol, were rapidly reduced, resulting in a color change to black, followed by the injection of an ethylene glycol solution of PVP. After removal of ethylene glycol and PVP excesses via centrifugation, TEM analysis (Figure 2C) showed large networks of small (<10 nm) crystals that began to aggregate shortly after washing. This confirms that the ethylene glycol is working as a reducing agent, as well as increasing the viscosity of the solution, thus contributing to the formation of the small nanocrystals that can combine into larger, polycrystalline NPs when the solvent is a mixture of ethylene glycol and water. Due to the difficulty of recovering particles from the more viscous solutions, continued testing was performed on alloys synthesized in a 75% ethylene glycol solution, with a diameter of approximately 20 nm (20 nm-Pd_7_Au_3_).

A key component of any alloyed metallic structure, including nanostructures, is the identification of the extent of alloying, including any potential core–shell formation or uneven distribution of the elements across the alloyed structure. These potential shells or defects can negatively affect the application of the alloy structure but are quite commonly observed and are often remedied through thermal annealing or other postsynthetic routines. For the PdAu system, the formation of a thin Au shell has been observed in some nanostructures, leading to the presence of hysteresis during hydrogen-sensing applications.^56,57^ Since the colloidal synthesis of our PdAu NPs is performed at room temperature, with minimal heating or other treatments to alter the atomic postsynthetic structure, it is crucial to determine the distribution of the metals within the NPs. We therefore extensively characterized the 20 nm-Pd_7_Au_3_ NPs through a variety of methods designed to assess the potential formation of an Au shell or other possible alloy defects, such as phase separation.

**Figure 3:**
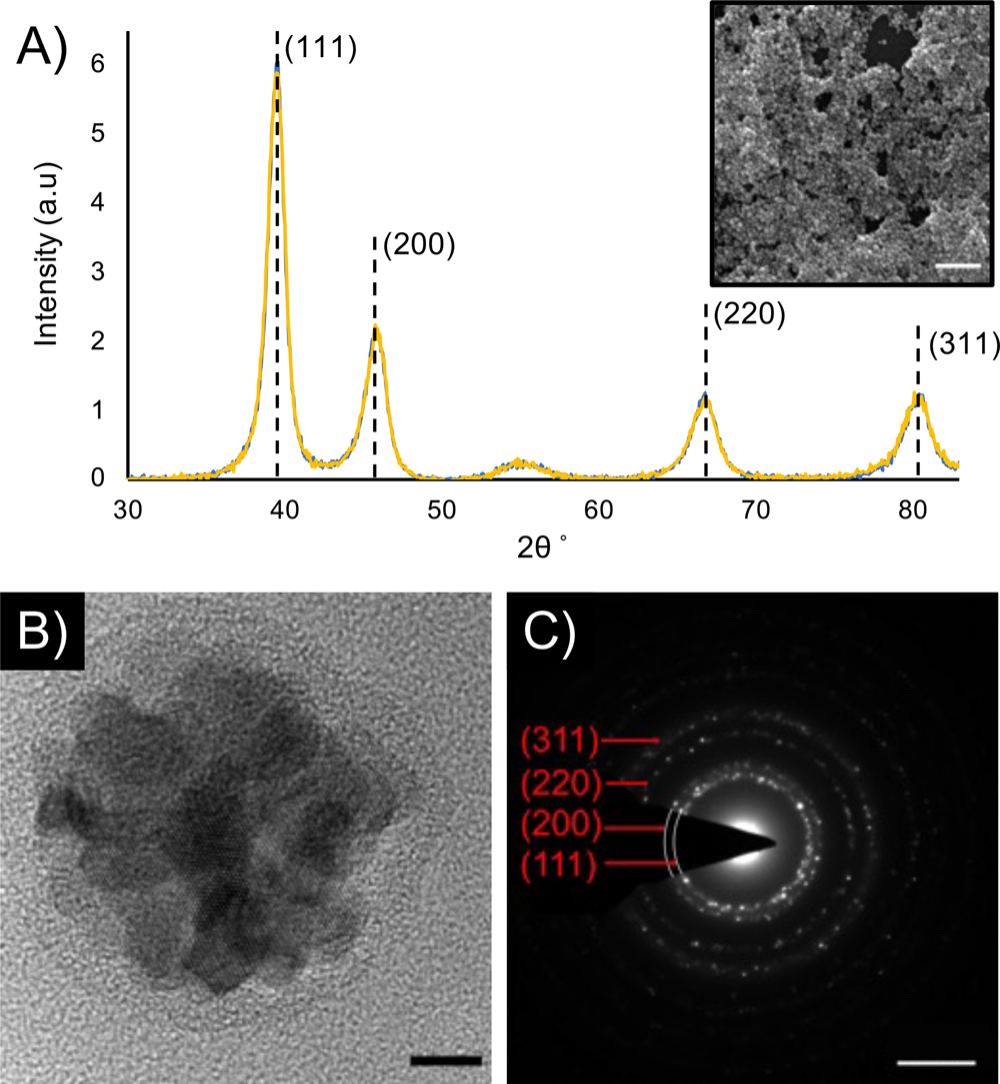
Characterization of alloy formation in 20 nm-Pd_7_Au_3_NPs. (A) GIXRD patterns for 20 nm-Pd_7_Au_3_NPs (SEM inset, scale bar is 200 nm) before (blue) and after four hydrogenation cycles (yellow), indicating homogeneous alloy formation and that no significant restructuring occurs during hydrogen sorption. The broad peak around 55° 2θ is the (311) reflection from the Si(100) wafer that served as a substrate. (B) HRTEM image of a single 20 nm-Pd_7_Au_3_NP, showing high polycrystallinity. The scale bar is 5 nm. (C) SAED pattern from 20 nm-Pd_7_Au_3_NPs, resulting in a lattice constant of 4.03 ± 0.03 Å. The scale bar is 5 nm^–1^.

Grazing-incidence XRD (GIXRD) experiments were performed to assess the average crystallite sizes and overall composition of the NPs and to exclude the formation of pure Au or Pd phases within NPs. One sample was additionally subjected to four hydrogenation cycles (600 mbar, 30 s) to investigate potential restructuring after hydrogen sorption. GIXRD results from both samples are included in Figure 3A, demonstrating the lack of changes in crystal structure or crystallite size after the hydrogenation treatment. The estimated lattice constant derived from Rietveld refinement is 3.96 ± 0.02 Å for both samples—before and after hydrogenation (Figure S7, SI). The fit gives a relatively good *R*-factor of 12.6%. Since Pd and Au both crystallize in face-centered cubic (fcc) lattices, there exists a linear relation between the lattice constant and the composition of the alloy (Figure S8, SI).^58^ On the basis of this linear relation, we can use these measurements to estimate the ratio of Pd:Au as 64.6% Pd and 35.4% Au, which is in good agreement with the elemental analysis results (Figure S5, SI) and the expected composition based on starting materials (Table S1, SI). Given the geometry of the setup and experimental approach (parallel beam, close to grazing incidence, nonmonochromatic beam), as well as crystallite sizes below 10 nm and the possible strain in the crystallites, we observe significant broadening of diffraction peaks in the patterns. The size of the crystallites was refined to 4.7 ± 1 nm. As TEM images indicate that each particle contains about four crystallites along its diameter, this value corroborates the microscopy results for the 20 nm-Pd_7_Au_3_ NPs.

Although the GIXRD does not indicate the formation of isolated areas of Au or Pd in the NPs and thus supports the formation of a homogeneous alloy, eliminating the possibility of significant core–shell formation or the formation of NPs of individual elements, single NP techniques using electron microscopy are useful to visualize the structure and investigate the formation of smaller shells that may fall outside the detection limits of GIXRD. HRTEM and SAED were therefore performed on 20 nm-Pd_7_Au_3_ NPs (Figure 3B,C), resulting in an estimated lattice constant of 4.03 ± 0.03 Å, which is slightly above the lattice constant determined from GIXRD (3.96 ± 0.02 Å) and the expected results from the linear relation between lattice constant and alloy composition for Pd–Au systems (3.95 ± 0.005 Å),^58^ which is due to the individual nature of this technique, as well as the size-dependent lattice expansion that has been observed in NPs.^59^ There is no indication of isolated Au or Pd crystallites/grains in the HRTEM or in the SAED patterns, indicating that while the PdAu NPs are quite polycrystalline, there is no significant phase separation.

**Figure 4:**
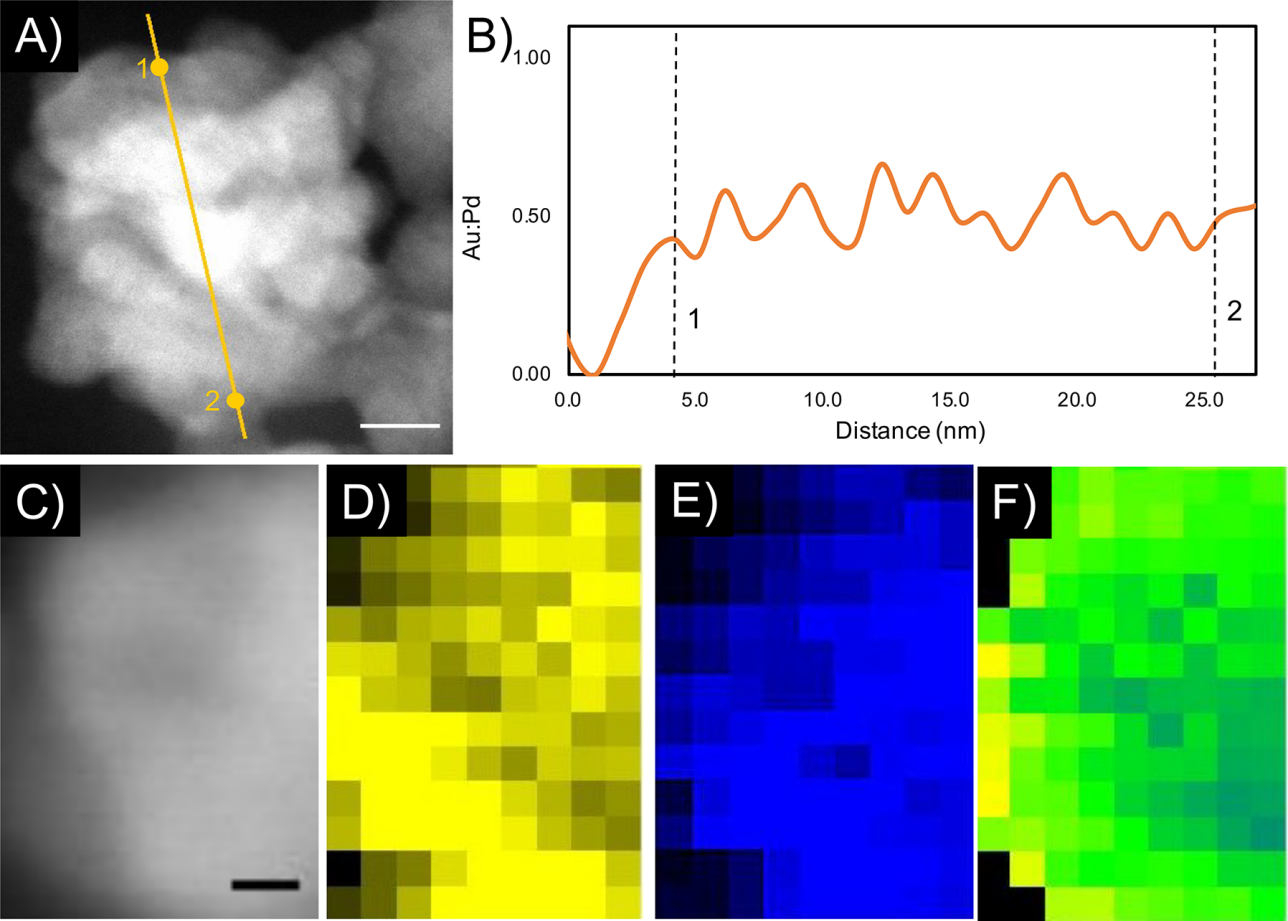
Characterization of alloy formation in PdAu NPs. (A) STEM image of 20 nm-Pd_7_Au_3_ NP with markers indicating the path of the electron beam for EDX analysis. The scale bar is 2 nm. (B) EDX analysis for a NP from part A plotted as ratio of Au:Pd. (C) STEM image of 20 nm-Pd_7_Au_3_ NPs used for EDX 2D mapping, demonstrating the effective alloying of NPs with different sizes and Pd:Au ratios. The scale bar is 2 nm. (D) EDX 2D map of Au (yellow). (E) EDX 2D map of Pd (blue). (F) Overlay of Au and Pd EDX 2D maps, showing lack of defined Au or Pd nanocrystal formation.

In addition to the SAED, we also used scanning TEM (STEM) in conjunction with EDX to determine Pd:Au ratios for individual NPs (Figure S9, SI) and to map profiles and images of multiple 20 nm-Pd_7_Au_3_ NPs to show the distribution of the metals across the NPs. As shown in Figures 4B and S10 (SI), a line scan across an individual NP does not feature any significant Au shell or, indeed, any significant local accumulation of Au or Pd within, for example, a single crystallite, which confirms the formation of a homogeneous alloy. Additional EDX scans before and after four hydrogenation cycles (600 mbar, 30 s) indicate no change in composition or atomic reconstruction after hydrogen sorption (Figure S11, SI). This conclusion is further corroborated by 2D EDX mapping of a 20 nm-Pd_7_Au_3_ NP (Figure 4C–F). Altogether, these findings convincingly confirm the synthesis of high-quality PdAu alloy NPs with the expected alloyant ratios.

**Figure 5:**
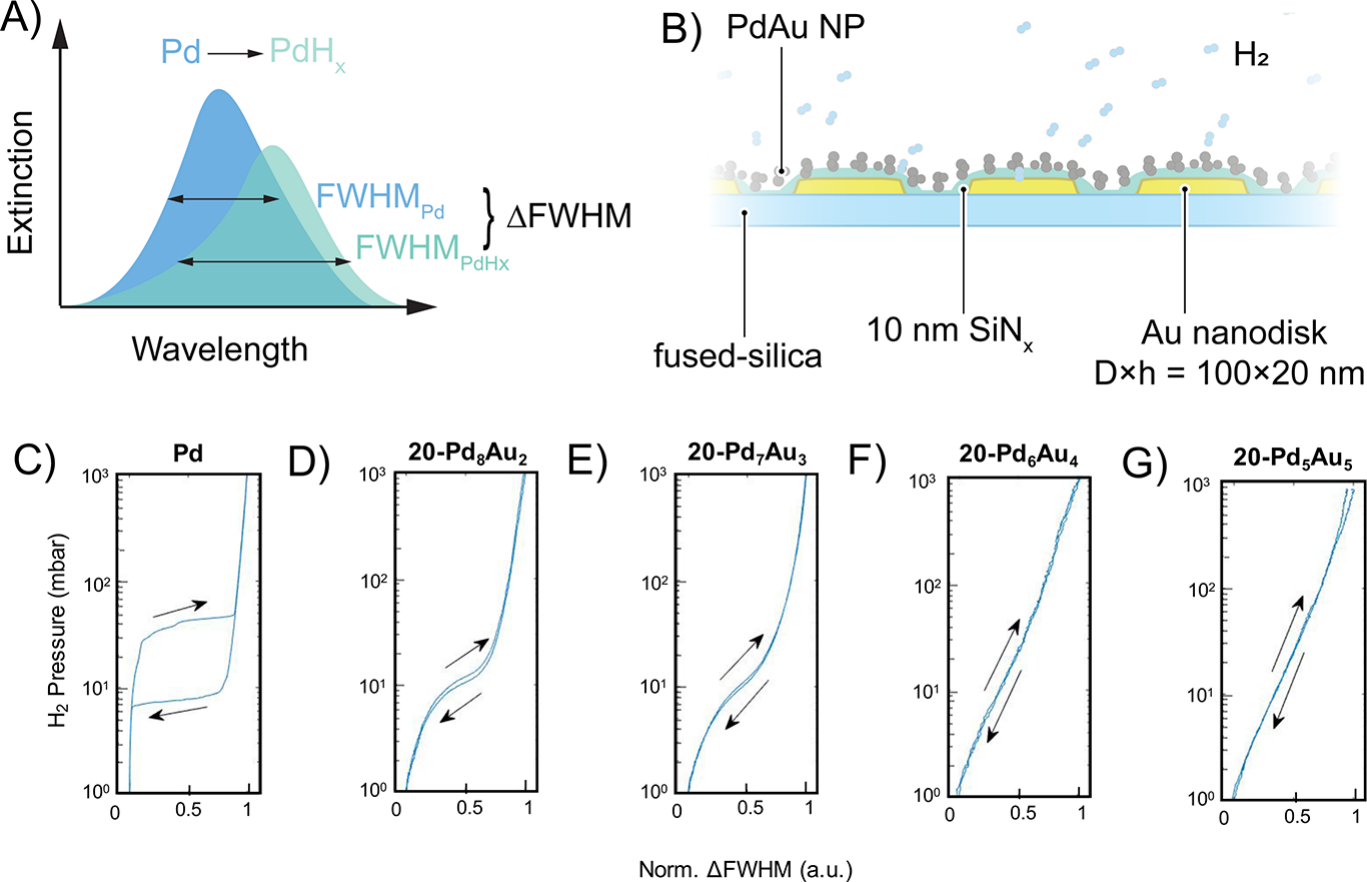
Hydrogen pressure–optical response isotherm measurements for PdAu alloy NPs. (A) Schematic of the indirect nanoplasmonic sensing (INPS) readout of the LSPR peak, where we used a shift in full width at half-maximum (fwhm) as the readout parameter. (B) Schematic of the INPS chips used for H_2_ sorption measurements, with the colloidal Pd or PdAu NPs deposited on nanolithographically patterned substrates comprising inert, SiN_*x*_-covered Au nanodisks as plasmonic probes, (C–G) Isotherms for 20 nm-Pd, 20 nm-Pd_8_Au_2_, 20 nm-Pd_7_Au_3_, 20 nm-Pd_6_Au_4_, and 20 nm-Pd_5_Au_5_ NPs, showing the dramatic effects of alloying on the hydrogen-sensing response, where it decreases both hysteresis and shrinks the width of the α–β transition plateau, as a consequence of a reduced critical temperature, *T*_C_.

To demonstrate a first application of our PdAu alloy NPs in a proof-of-principle fashion, we evaluated their performance as hysteresis-free hydrogen sensors.^33^ Specifically, we used the 20 nm-PdAu NPs to confirm that they can replicate the key traits of similar systems made by lithography-based nanofabrication, which were investigated in previous works.^22,33,60^ Since the plasmon peak of our 20 nm-PdAu NPs lies near the limit of spectrometers optimized for visible light, we employed the indirect nanoplasmonic sensing (INPS)^61,62^ approach for their characterization. The INPS chip consists of a quasi-randomarray of Au nanodisks of diameter × height = 100 × 20 nm^2^, with a plasmon peak that lies in the visible spectral range (Figure 5B). These chips enable the measurement of hydrogen sorption in the alloy NPs deposited on top, via the tracking of spectral changes in the LSPR peak of the Au nanodisks that act as inert “observers”. The measured signal is the consequence of the change in local refractive index around the Au disks, induced by the hydrogen sorption in the 20 nm-PdAu NPs deposited on top.^61,62^ In this way, the Au nanodisks serve two purposes: (1) they enable the isotherm measurement in the visible spectral range (the 20 nm-PdAu NPs resonate in the UV due to their small size), and (2) they increase the signal-to-noise ratio in our measurement, which is intrinsically low due to the small optical cross sections of our 20 nm-PdAu NPs.^63^ Here, we also note that for future practical sensor applications, these two problems can be overcome by dispersing the PdAu alloy NPs in a polymer matrix, as we recently have demonstrated for colloidal Pd NP-based hydrogen sensors.^46,64^ In this way, the high refractive index of the polymer matrix will red-shift the LSPR and the larger number of NPs in the optical path due to 3D dispersion will mitigate the issue of the small optical cross sections of the individual particles.

For the isotherm experiments, we drop-cast a suspension of 20 nm-PdAu NPs with four different compositions (20 nm-Pd_8_Au_2_, 20 nm-Pd_7_Au_3_, 20 nm-Pd_6_Au_4_, and 20 nm-Pd_5_Au_5_), as well as a neat, PVP-stabilized Pd control (18 ± 6 nm, Figures S12 and S13, SI), onto the chips^44^ (Figure 5B). Using these INPS chips, we measured optical hydrogen absorption and desorption isotherms at 30 °C, using changes in the LSPR full width at half-maximum (Δfwhm) as a readout signal. It is worth noting that the optical response (i.e., Δfwhm) is proportional to the H/Pd ratio.^65^ Therefore, the pressure–optical response isotherm is equivalent to a “traditional” pressure–composition isotherm. As expected for the pure Pd control, we found a first-order phase transition (indicated by a distinct two-phase coexistence plateau) and significant hysteresis between the absorption and desorption branches (Figure 5C). The first-order transition is accompanied by lattice strain that is induced by the accomodation of hydrogen atoms on interstitial sites of the Pd host and is the reason for the two branches in the isotherm, known as hysteresis. Repeating the same measurements for increasing Au concentration in the alloy NPs then reveals the anticipated shrinking of the hysteresis gap until it completely vanishes for the 20 nm-Pd_6_Au_4_ sample (Figure 5D–G). The required Au concentration to suppress the hysteresis is similar to what has been previously reported for PdAu alloy nanostructures or thin films, which is around 30 wt %.^66,67^ The hysteresis suppression is the consequence of the larger Au atoms (compared to Pd), which minimizes lattice strain induced by hydrogen absortion into the lattice.^68^ Along with the material characterization discussed earlier, the pressure–optical response isotherms confirm homogeneous alloy formation throughout individual NPs because complete hysteresis suppression in the PdAu system would not be attained if the Au were not dispersed uniformly.^34,69^ However, small variations in the PdAu ratio between individual particles cannot be excluded. Upon even further increasing the Au concentration in the alloy, the change in the optical response (i.e., Δfwhm) becomes very small, since hardly any hydrogen will be absorbed due to the low Pd content in the system (Figures S14 and S15, SI).^22,24,33,60^ Furthermore, at the highest Au concentrations, the material (and thus the optical response) becomes less stable during the hydrogen absorption/desorption cycling. This is manifested by the nonclosing absorption/desorption branches at high pressure in the Pd_5_Au_5_ sample isotherm (Figure 5G). Therefore, we advocate Pd_6_Au_4_ as the most optimized composition, since the sensing response is hysteresis-free but remains stable upon hydrogen cycling.

## Conclusion

Here, we have demonstrated a robust, room-temperature, colloidal synthesis of polycrystalline PdAu alloy NPs of varying sizes and alloy compositions using ethylene glycol and water as a solvent mixture. The composition of these NPs can be easily adjusted during the synthesis, resulting in a wide variety of colloidal NPs. In addition, XRD, HRTEM, SAED, and EDX were used to confirm homogeneous alloy formation. Furthermore, we investigated the interaction of the synthesized PdAu alloy NPs with hydrogen gas and found the anticipated suppression of hysteresis at an Au concentration of 40%. In a wider perspective, the synthesis presented here potentially allows for the consistent, larger scale production of PdAu alloy NPs for hydrogen-sensing applications, e.g., through incorporation in a bulk-processed polymer matrix, as we recently have demonstrated.^46,64^ PdAu alloys are also applicable in the field of catalysis, for instance in electrocatalysis and as an effective catalyst for nitrite reduction.^15,17^ This only confirms that our approachable synthesis method opens up a possibility for making and designing PdAu alloys for a wide range of applications.

## Supporting Information Available

Elemental analysis of samples; additional synthesis details; theoretical predictions for alloy lattice constants; additional SEM, TEM, STEM, and EDX; and raw spectra from hydrogen pressure–optical response isotherms (PDF) (PDF)
