# Supplementary material for: Robust Colloidal Synthesis of Palladium–Gold Alloy Nanoparticles for Hydrogen Sensing
Source: ACS Appl Mater Interfaces. 2021 Sep 20;13(38):45758–67. doi: 10.1021/acsami.1c15315 (PMC8485326; doi:10.1021/acsami.1c15315)
Supplement: Supplementary file 1 — am1c15315_si_001.pdf [file am1c15315_si_001.pdf]

Supporting Information

Robust Colloidal Synthesis of Palladium-Gold Alloy Nanoparticles for Hydrogen Sensing

Sarah Lerch[a, †], *Alicja Stolaś*[a, †], *Iwan Darmadi*[b], *Xin Wen*[a], *Michał Strach*[b, c], *Christoph Langhammer*[b, *], *Kasper Moth-Poulsen*[a, *]

[a.] Department of Chemistry and Chemical Engineering, Chalmers University of Technology, SE-412-96 Gothenburg, Sweden.

[b.] Department of Physics, Chalmers University of Technology, SE-412-96 Gothenburg, Sweden.

[c.] Chalmers Materials Analysis Laboratory, Chalmers University of Technology, SE-412-96 Gothenburg, Sweden.

Corresponding authors:

*Kasper Moth-Poulsen – Department of Chemistry and Chemical Engineering, Chalmers University of Technology, SE-412 96 Gothenburg, Sweden.

Email: kasper.moth-poulsen@chalmers.se

*Christoph Langhammer – Department of Physics, Chalmers University of Technology, SE-412 96 Gothenburg, Sweden. Email: clangham@chalmers.se

Contents | Page

S1. Colloidal nanoparticle synthesis 3

Order of addition for reactants 4

Changing ratio of metallic salts 5

Addition of excess halide ions 6

Changes in physical conditions 7

Use of ethylene glycol / water mixtures as solvents 8

S2. X-ray powder diffraction in grazing incidence (GIXRD) / scanning electron microscopy (SEM) 9

Theoretical predictions for alloy lattice constants 10

S3. Transmission electron microscopy (TEM) / selected area electron diffraction (SAED) / energy-dispersive x-ray spectroscopy (EDX) 11

S4. Hydrogen pressure-optical response isotherms 14

References 17

S1. Colloidal nanoparticle synthesis

Centrifugation was performed with a Hereus Multifuge X1 with a Fiberlite F15-8 x 50cy Fixed Angle Rotor.

Elemental analysis was performed by Mikroanalytisches Labor Kolbe c/o Fraunhofer Institut UMISCHT.

TEM images in this section, S1, were obtained on a FEI Tecnai T20 with a LaB6 filament and Orius CCD, operated at 200 kV. TEM samples were prepared by drying a 3-5 μL drop of colloidal NP solution, in air, on a copper TEM grid with carbon film (200 mesh, Ted Pella).

Desired Synthesis Ratio (% Pd / %Au)	Desired Synthesis Ratio (mmol Pd / mmol Au)	Na_2PdCl_4 (g, ±0.001)	$HAuCl_4$ (g, ±0.001)
90 / 10	18 / 2	0.053	0.008
80 / 20	16 / 4	0.047	0.016
70 / 30	14 / 6	0.041	0.024
60 / 40	12 / 8	0.035	0.032
50 / 50	10 / 10	0.029	0.039
30 / 70	6 / 14	0.018	0.055
10 / 90	2 / 18	0.006	0.071

Table S1 Amount of metallic salts used for PdAu alloy NP synthesis, reported as ratios, in % and molar values, and as mass of each metallic salt, in g.

Order of addition of reactants

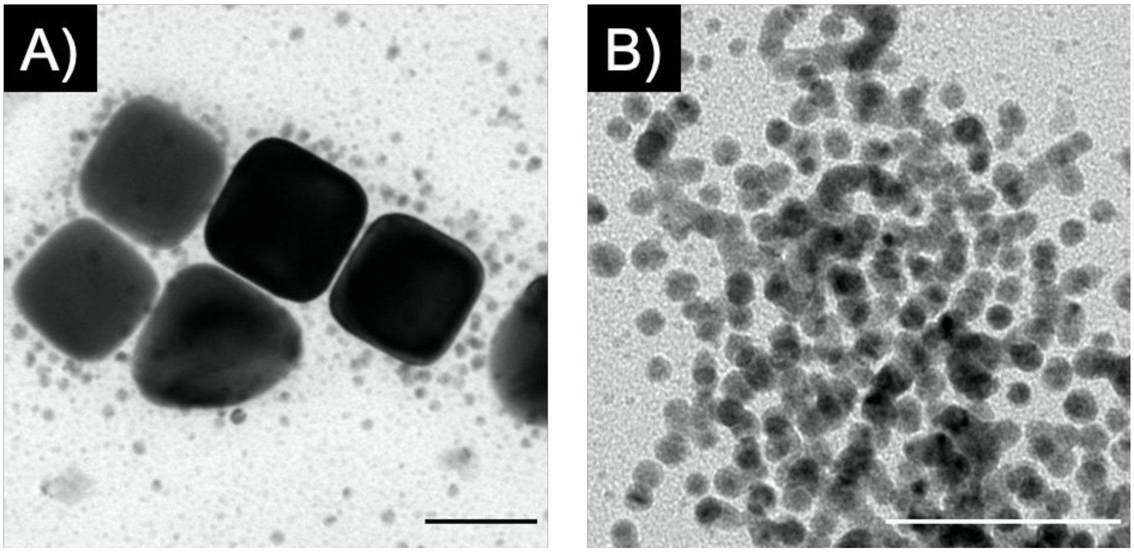

Figure S1 In order to confirm the importance of adding the reducing agent (ascorbic acid) prior to the stabilizer (PVP), we repeated the synthesis as above but added the PVP prior to the ascorbic acid **(A)** and added both reactants at the same time **(B)**. Both showed significant differences from the original alloy synthesis, resulting from the isolation of the individual metals by the PVP molecules. Scale bars = 50 nm.

Changing ratio of metallic salts

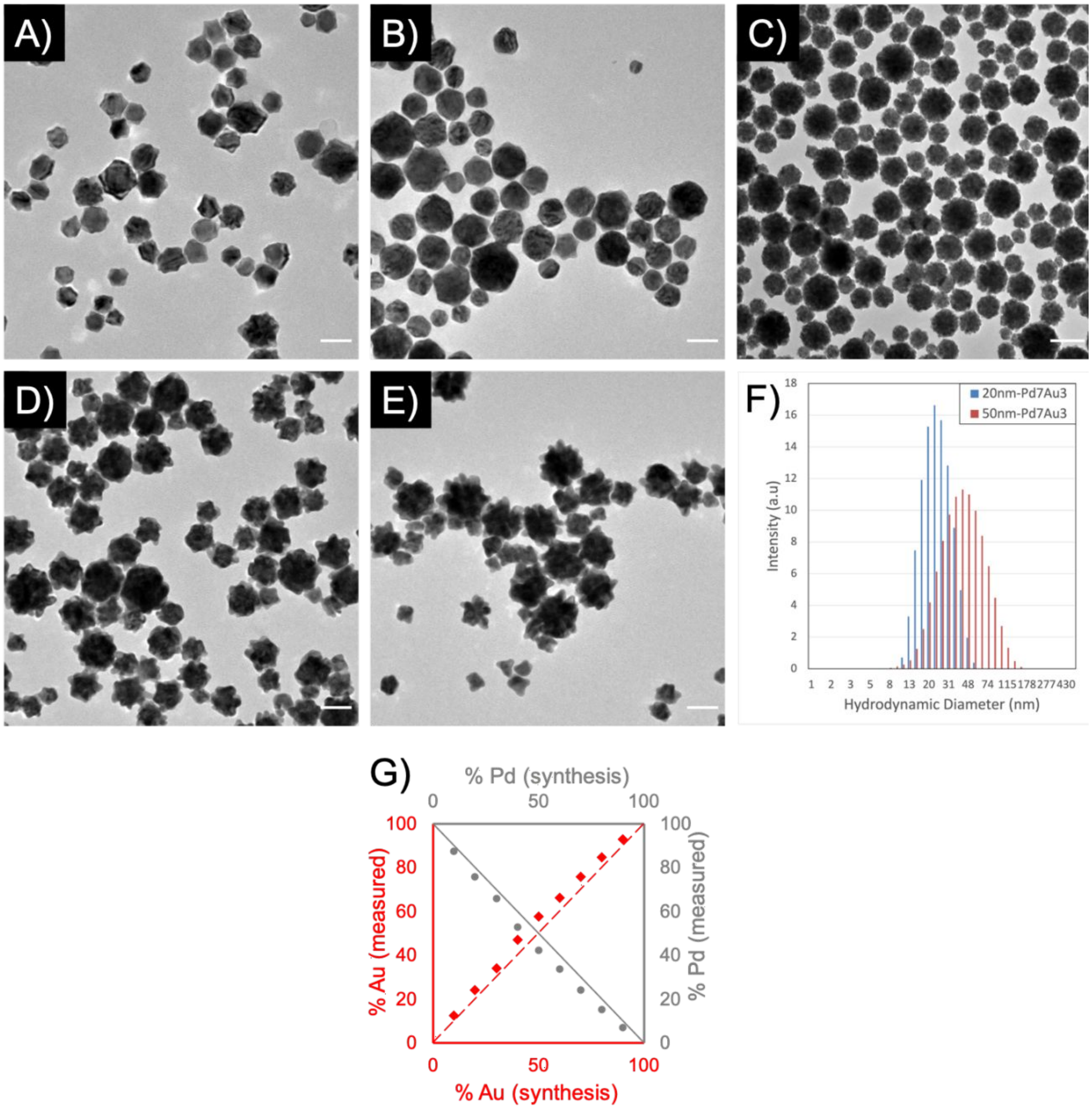

Figure S2 This synthesis method is applicable for a range of Pd:Au ratios from 90% Pd, 10% Au (Pd_9Au_1) to 10% Pd, 90% Au (Pd_1Au_9), nearly encompassing the full range of potential ratios that can be desired from PdAu alloy NPs. Here we show a selection of these ratios for NPs with an average size of 47 ± 14 nm, including Pd_9Au_1 **(A)**, Pd_7Au_3 **(B)**, Pd_5Au_5 **(C)**, Pd_3Au_7 **(D)**, and Pd_1Au_9 **(E)**. Scale bars = 50 nm. **(F)** Size of solution from (B, red) and 20nm-Pd_7Au_3 (blue, see Figure 2) as measured using dynamic light scattering (DLS). **(G)** Results from elemental analysis, demonstrating that the experimentally measured Au (red) and Pd (gray) percentages (x-axes) approximately match the theoretical

percentages as introduced in the synthesis (y-axes), although the Au content is slightly higher due to the faster reduction of Au^{3+} when compared to Pd^{2+}.

Addition of excess halide ions

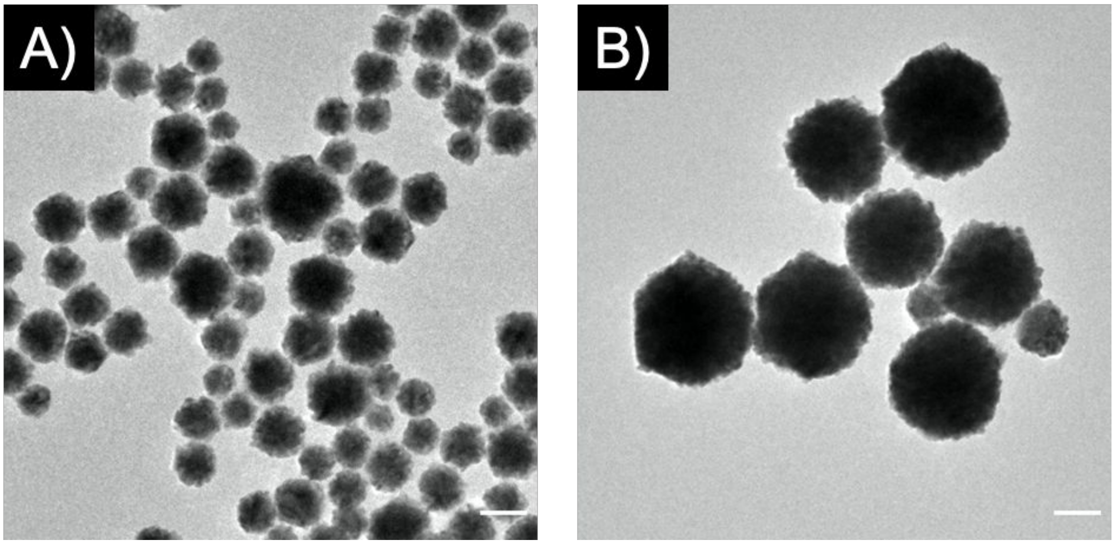

Figure S3 Due to significant interest in the control of surface facets on NPs, we attempted to control the surface facets through the addition of halide ions, Br^{-} and Cl^{-} ions through the use of NaBr **(A)** and KCl **(B)**, respectively. Shown here, neither halide ion contributed to significant shape changes in the NPs. Scale bars = 50 nm.

Changes in physical conditions

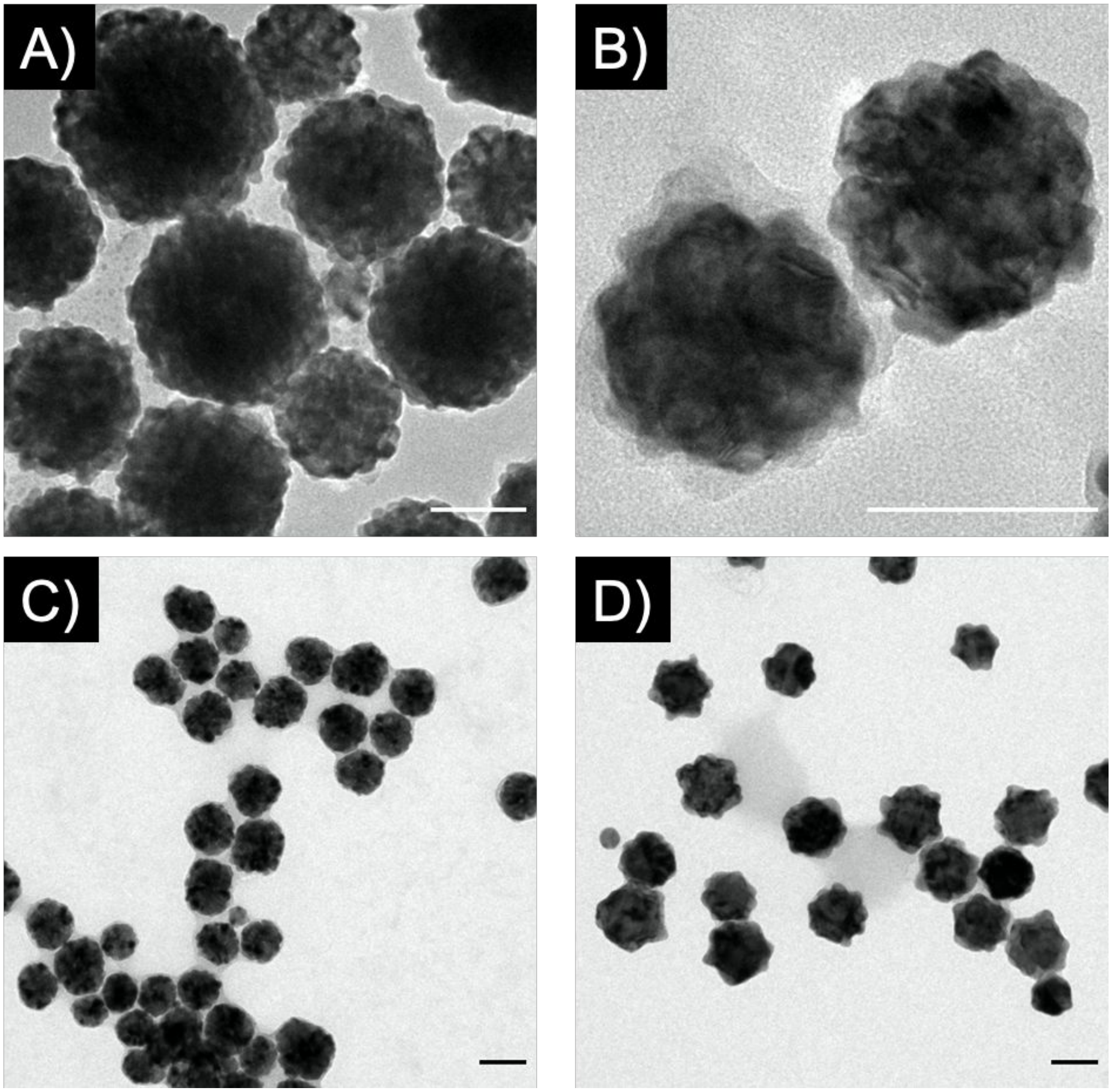

Figure S4 Increasing the stirring speed from 500 rpm to 750 rpm **(A)** or 1000 rpm **(B)** did not result in significant changes to the size of the NPs. Additionally, changes to the temperature, both decreasing from room temperature (20˚C) to 0˚C **(C)** and increasing to 50˚C **(D)**, also did not result in significant changes to the NPs. Scale bars = 50 nm.

Use of ethylene glycol / water mixtures as solvents

The use of an ethylene glycol / water as the solvent for the NP reduction and growth contributes to an increased viscosity as well as an additional reducing agent to aid in the formation of small PdAu alloy NPs with a good size distribution. The synthesis is repeated as before, with two minor changes. The L-ascorbic acid is added as described above and then the PVP is added after the solution changes color, a process which can take up to 2 minutes (90% ethylene glycol). Additionally, the NPs are washed with acetone (50 mL, doubling the total solution volume) and centrifuged at 10 000 for 1 hour to effectively collect the smaller NPs from the viscous solution. Here, we show the effect of increasing the volume % of ethylene glycol in the solvent mixture for Pd_7Au_3 alloy NPs (Figure S1.5), as well as the variety of 20 nm NPs, deposited in a densely packed monolayer, that were used for optical-pressure composition isotherms (Figure S4.1).

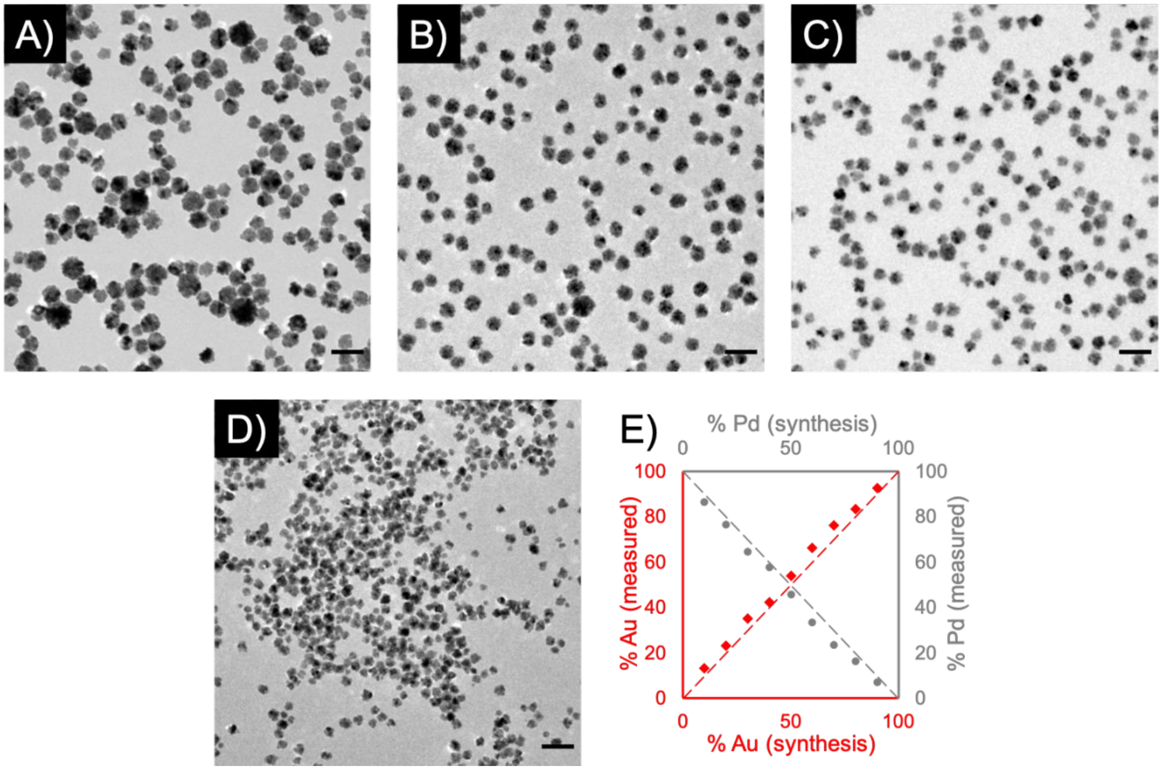

Figure S5 The NPs synthesized in 25% ethylene glycol / 75% water **(A)** are 28 ± 7 nm in diameter, while NPs synthesized in 50% ethylene glycol / 50% water **(B)** are 24 ± 4 nm in diameter. Increasing further to 75% ethylene glycol / 25 % water **(C)** and 90% ethylene glycol / 10 % water **(D)** are also included in the main text (Figure 2A,B). Scale bars = 50 nm. **(E)** Results from elemental analysis, demonstrating that the experimentally measured Au (red) and Pd (gray) percentages (x-axes) approximately match the theoretical percentages as introduced in the synthesis (y-axes), although the Au content is slightly higher due to the faster reduction of Au^{3+} when compared to Pd^{2+}.

S2. X-ray powder diffraction in grazing incidence (GIXRD) / scanning electron microscopy (SEM)

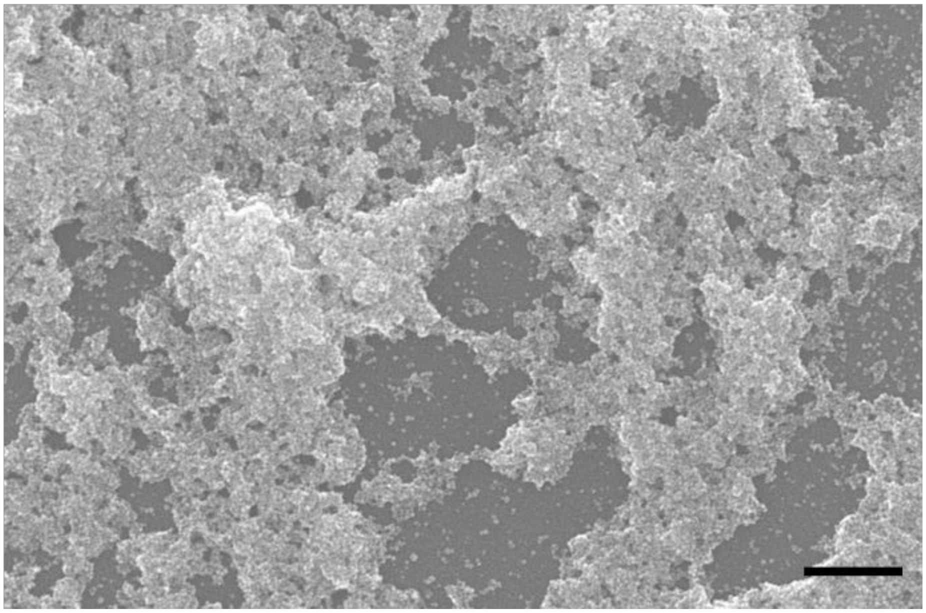

Figure S6 SEM image of 20nm-Pd_7Au_3 alloy NPs deposited on Si chip for GIXRD measurements. Scale bar = 500 nm.

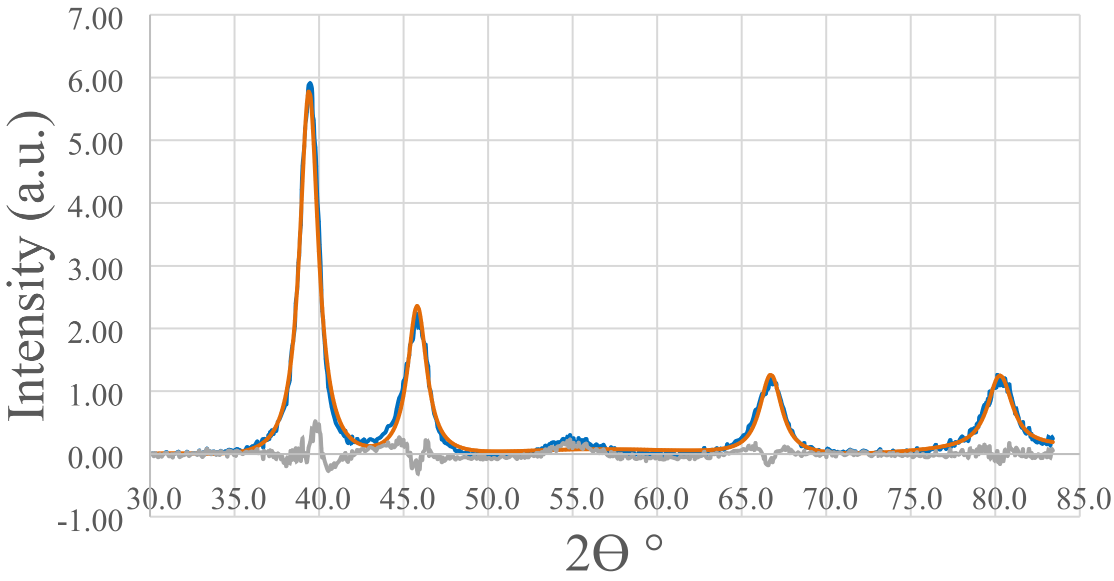

Figure S7 Rietveld refinement (orange) for GIXRD results from the sample after hydrogenation (blue), including the noise variation (grey). Specifics of the software and corrections used are included in the main text experimental section.

Theoretical predictions for alloy lattice constants

Pd is completely miscible in Au, as both crystallize in the face-centered cubic (fcc) lattices, and there exists a linear relation between the lattice constant and composition (Figure S2.2).[1]

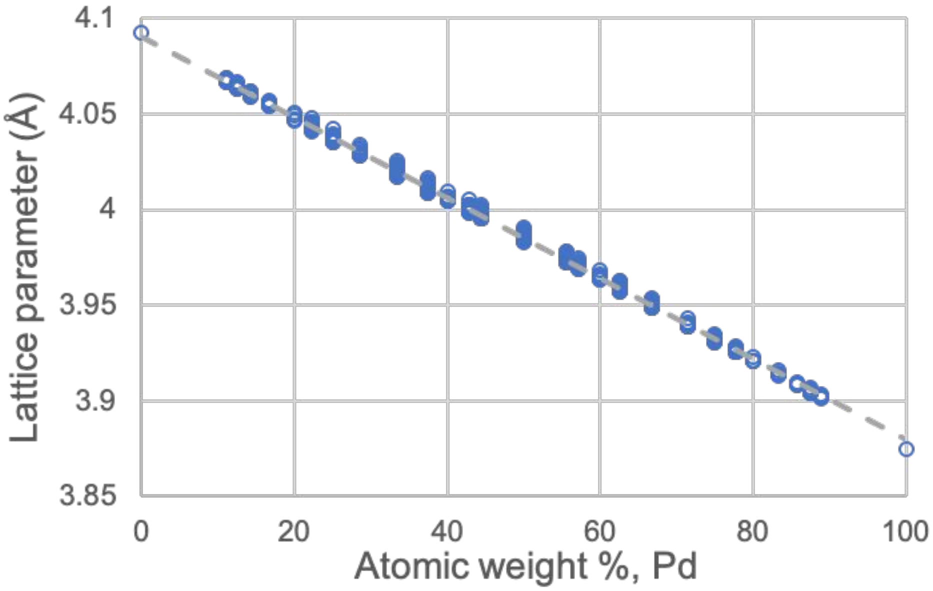

Figure S8 Trend of lattice parameter vs. composition for the Pd-Au binary alloy system.

S3. Transmission electron microscopy (TEM) / selected area electron diffraction (SAED) / scanning-transmission electron microscopy (STEM) / energy-dispersive x-ray spectroscopy (EDX)

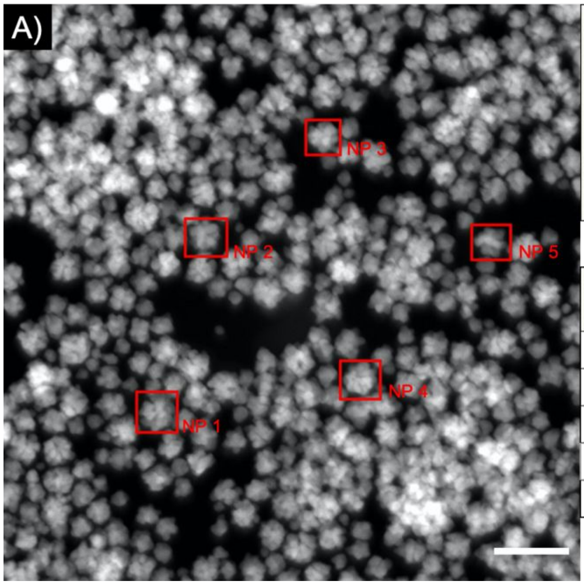

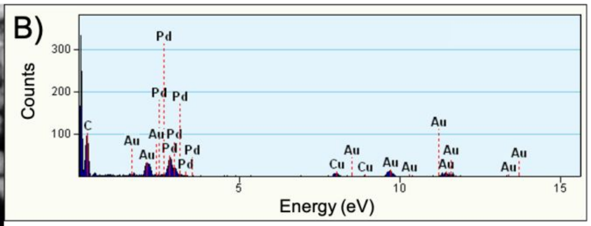

C)

EDX Area	% Pd	% Au	% Other (Cu + C)	Adjusted % Pd	Adjusted % Au
NP 1	5.44	2.36	92.18	69.7	30.3
NP 2	7.04	2.61	90.33	73.0	27.0
NP 3	5.81	2.14	92.03	73.1	26.9
NP 4	5.01	1.98	92.98	71.7	28.3
NP 5	4.30	1.78	93.88	70.7	29.3

Figure S9 STEM imaging and EDX measurements of individual 20nm-Pd_7Au_3 NPs. **(A)** STEM image of large area of NPs where the outlined areas were measured with EDX to confirm average %Pd and %Au for multiple NPs. **(B)** Sample EDX pattern for NP 1, with Pd, Au, Cu and C marked as the primary elements identified. Cu and C are present in the TEM grid and support film, respectively. **(C)** Raw atomic % for Pd, Au and other elements combined (primarily Cu and C) as well as atomic % for Au and Pd adjusted for direct comparison.

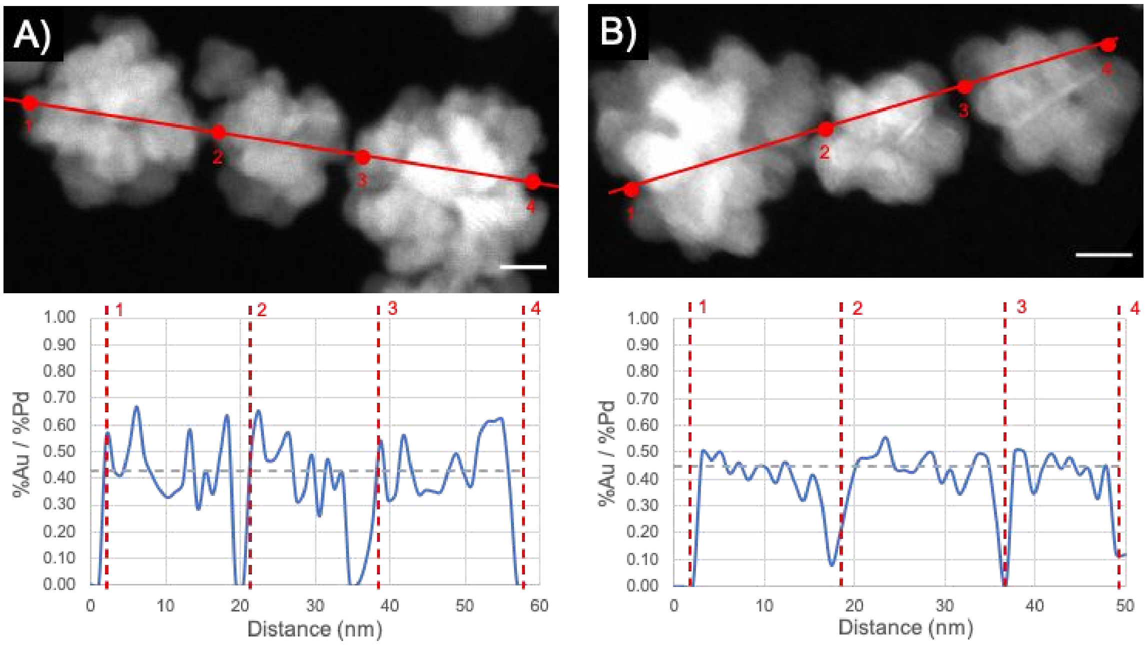

Figure S10 STEM imaging and EDX profile measurements of individual 20nm-Pd_7Au_3 NPs. **(A)** STEM image (top) of three 20nm-Pd_7Au_3 NPs where the red line indicates the line of the EDX profile scan (bottom, blue, solid line) with identifying points marked on scan (red dashed line). Gray dashed line indicates average Au:Pd ratio, corresponding to particles of approximately 70% Pd, 30% Au. **(B)** STEM image (top) of three 20nm-Pd_7Au_3 NPs where the red line indicates the line of the EDX profile scan (bottom, blue, solid line) with identifying points marked on scan (red dashed line). Gray dashed line indicates average Au:Pd ratio, corresponding to particles of approximately 69% Pd, 31% Au.

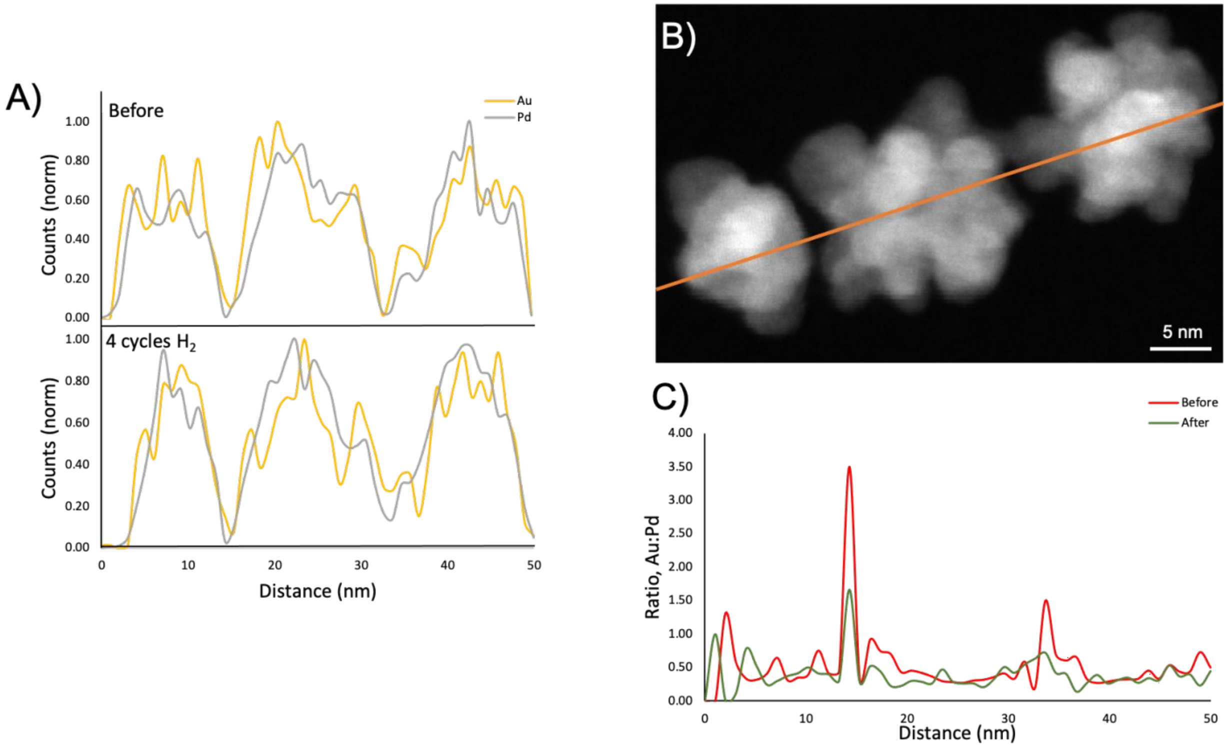

Figure S11 STEM imaging and EDX profile measurements of 20nm-Pd_7Au_3 NPs before and after hydrogenation, indicating relatively little change in composition after hydrogenation. **(A)** EDX profile scans over 3 NPs for Au (yellow) and Pd (gray). Top profile scan is prior to hydrogenation and bottom profile is after 4 cycles of hydrogenation (600mbar, 30 seconds per cycle). **(B)** STEM image of three 20nm-Pd_7Au_3 NPs where the orange line indicates the line of the EDX profile scans shown in A. **(C)** EDX profile scan over same three NPs, before (red) and after (gren) hydrogenation cycles, adjusted as the ratio between Au:Pd.

S4. Hydrogen pressure-optical response isotherms

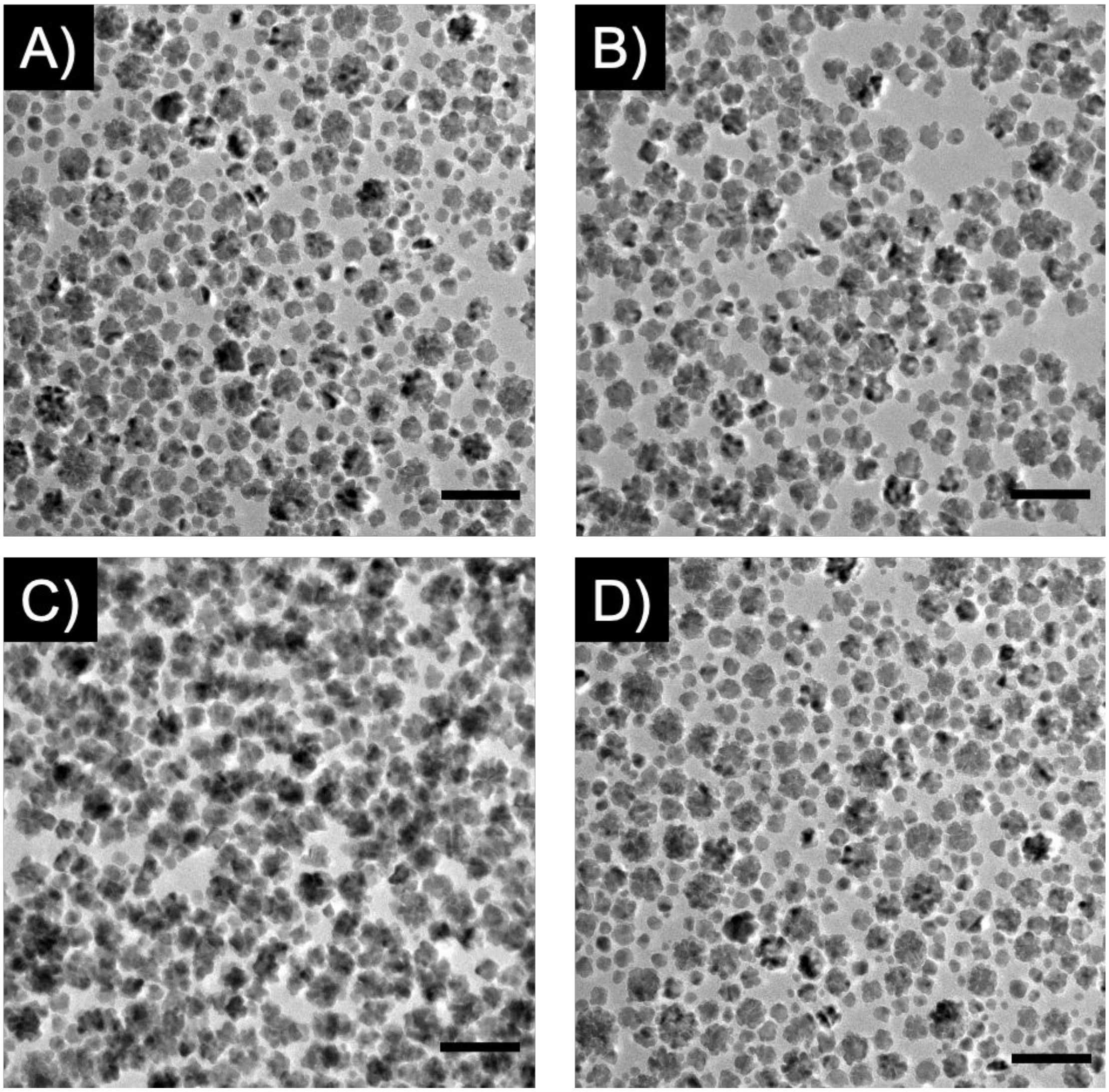

Figure S12 Other ratios of PdAu alloy NPs were synthesized with a solvent mixture of 75% ethylene glycol / 25% water for optical-pressure composition isotherms. Shown here are densely packed monolayers for 20nm-Pd_8Au_2 **(A)**, 20nm-Pd_7Au_3 **(B)**, 20nm-Pd_6Au_4 **(C)**, and 20nm-Pd_5Au_5 **(D)**. Additional images for 20nm-Pd_7Au_3 are included above in S1.5C as well as in the main text (Figure 2A). Hydrogen pressure-optical response isotherms are included in the main text (Figure 5D-F, 20nm-Pd_8Au_2, 20nm-Pd_7Au_3, 20nm-Pd_6Au_4) and spectra are included in section S5, below. Scale bars = 50 nm.

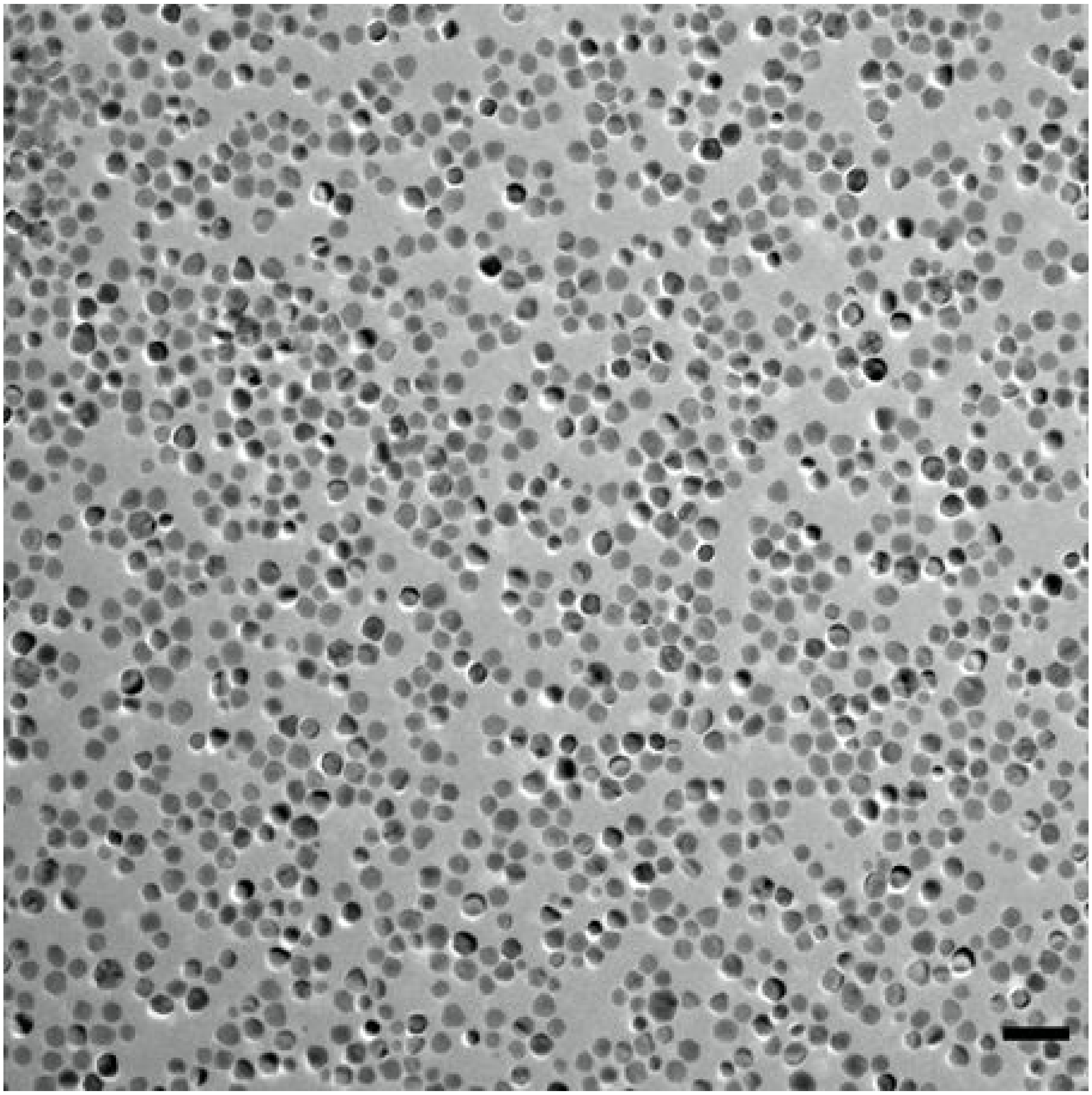

Figure S13 PVP-stabilized Pd NPs with an average diameter of 18 ± 6 nm, synthesized as described above. Scale bar = 50 nm.

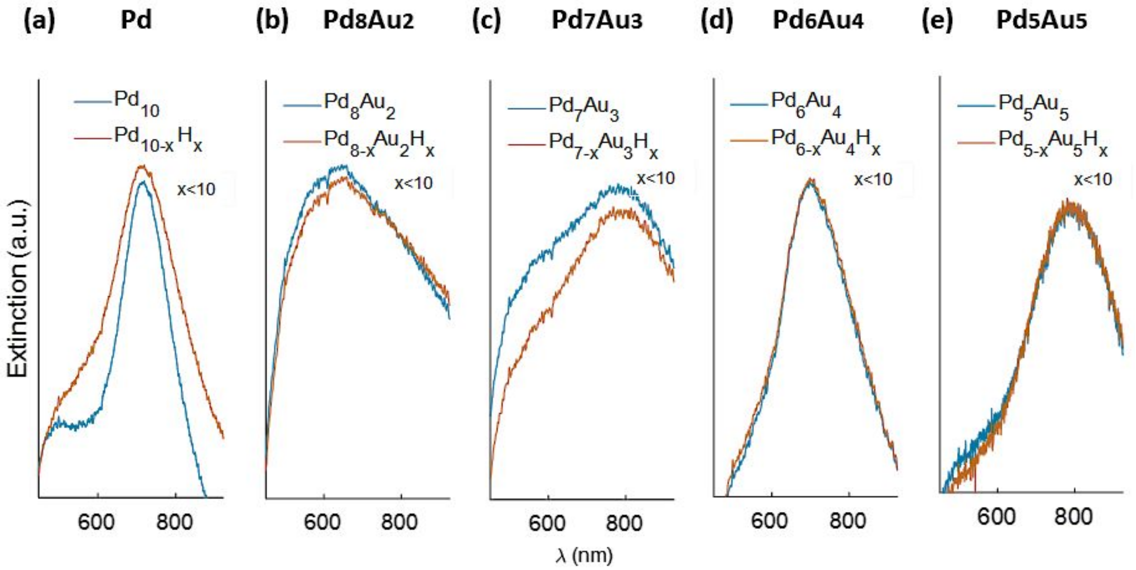

Figure S14 Raw spectra used to construct hydrogen pressure-optical response isotherms (Figure 5c) for **(A)** 20nm-Pd, **(B)** 20nm-Pd_8Au_2, **(C)** 20nm-Pd_7Au_3, **(D)** 20nm-Pd_6Au_4, and **(E)** 20nm-Pd_5Au_5. Two representative spectra, namely non-hydrated Pd (alloy) and hydrated Pd (alloy) at c.a. 1000 mbar, are shown in blue and red lines, respectively. The spectra were obtained at 30˚C.

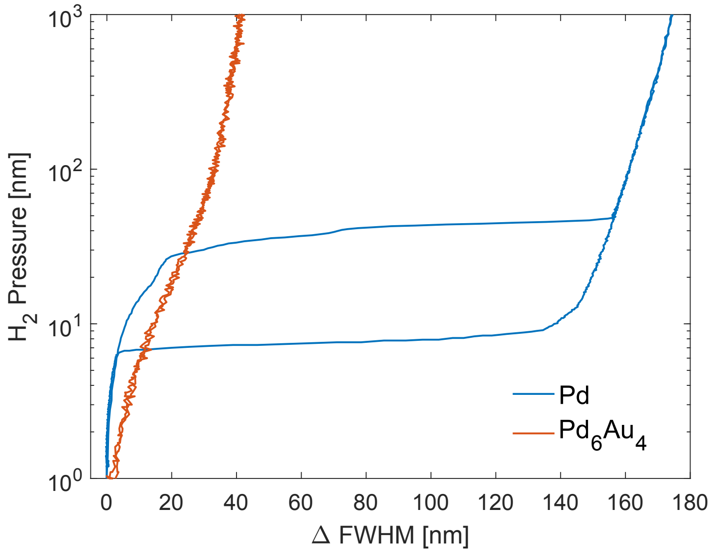

Figure S15 Comparison of Pd and Pd_6Au_4 hydrogen pressure-optical response (ΔFWHM, in nm) isotherms obtained at 30˚C. Note the difference of the overall optical contrast upon hydrogenation between the two samples.

References

(1) Okamoto, H.; Massalski, T. B. The Au− Pd (Gold-Palladium) System. *J. Phase Equilibria* **1985**, *6* (3), 229–235.
